# Endoscopic transorbital approach for recurrent spheno-orbital meningiomas: single center case series

**DOI:** 10.1007/s10143-024-02905-z

**Published:** 2024-09-30

**Authors:** V. Ricciuti, E. Peppucci, A. Montalbetti, G. Piras, G. Spena, C. G. Giussani, C. Zoia

**Affiliations:** 1 School of Medicine and Surgery, University of Milano-Bicocca, Monza, 20900 Italy; 2 Neurosurgery, Fondazione IRCCS San Gerardo dei Tintori, 20900 Monza, Italy; 3 UOC of Neurosurgery, Ospedale Moriggia Pelascini, Gravedona e Uniti, 22015 Gravedona, Italy; 4 Neurosurgery Unit, Fondazione I.R.C.C.S. Policlinico San Matteo, Pavia, Italy

**Keywords:** Endoscopic transorbital approaches, Recurrent spheno-orbital meningiomas, Spheno-orbital meningiomas, Neuro-oncology

## Abstract

Endoscopic transorbital approaches (ETOAs) are finding wide application for skull base lesions, particularly for spheno-orbital meningiomas (SOMs). These tumors have high recurrence rates, and second surgery can often represent a challenge. In this study we analyze our experience of management of recurrent SOMs through a slightly modified eyelid crease approach. Between May 2016 and September 2023, in the Department of Neurosurgery of Fondazione IRCCS Policlinico San Matteo (Pavia, Italy), five consecutive recurrent SOMs have been treated using an endoscopic transorbital approach. Demographic data, preoperatory deficits, lesions characteristics, histology, grade of resection, eventual adjuvant treatments, complications, outcome in terms of symptoms improvement and cosmesis, and hospitalization are described. One patient maintained a right lateral rectus muscle palsy that was already present in the preoperatory, no cerebrospinal fluid (CSF) leaks were reported. All patients had postoperative periorbital edema, but no other systemic complication was found. All patients had proptosis improvement, two had visual acuity improvement, and best cosmetic outcome was obtained in all cases. Hospitalization varied between 4 and 6 days. ETOAs in the management of recurrent SOMs are safe and have good outcome. Right selection of patients is mandatory, but when feasible, endoscopic surgery can allow a virgin route to a previously operated tumor, guaranteeing a good strategic option.

## Introduction

Endoscopic transorbital approaches have been increasingly used in the last few years. Starting in the 80s when they first were proposed, they gathered more enthusiasm in 2010 when Moe et al. standardized the term transorbital neuroendoscopic surgery (TONES) [20], and now they are finding wider application and indication, also because they can be used in combination with endoscopic transnasal or open surgical approaches [30].

A growing literature is describing the application of endoscopic transorbital approaches for lesions of the middle cranial fossa, especially for those involving the lateral aspect of the sphenoid wing, but also for the cavernous sinus and the optic nerve or foramen [5, 7, 10, 29]. This is particularly true for spheno-orbital meningiomas (SOMs) [11]. These lesions have been traditionally managed with microsurgical transcranial approaches (MTAs), such as pterional or frontotemporal-orbitozygomatic craniotomies and their variants, but recentely endoscopic endonasal (EEAs), endoscopic transorbital (ETOAs) and combined approaches have been developed and used [18]. Giving their recent application, there are few articles that describe the use of ETOAs in the management of SOMs, even if growing numbers are demonstrating good safety and efficacy of these techniques [2, 8, 15, 17]. Another issue in the management of SOMs is the high rate of recurrence, that is up to 24.8% (with all surgical techniques) [1]. We believe that recurrent SOMs can represent a challenge, especially when there is an involvement of the orbit. In this article we describe our experience of five cases of recurrent lesions treated with a slightly modified superior eyelid crease approach. To our knowledge, while recurrent SOMs and MTAs have been described [19], this is the first article that focuses on exclusively recurrent SOMs treated with an endoscopic transorbital approach, and we believe that in selected cases, great results can be achieved through this technique.

## Materials and methods

Between May 2016 and September 2023, in the Department of Neurosurgery of Fondazione IRCCS Policlinico San Matteo (Pavia, Italy), five consecutive recurrent SOMs have been treated using an endoscopic transorbital approach. We described a surgical series in which we retrospectively reviewed demographic data, preoperatory deficits, lesions characteristics, histology, grade of resection, eventual adjuvant treatments, complications, outcome in terms of symptoms improvement and cosmesis, and hospitalization. We included all patients with recurrent SOMs older than 18 y.o. and excluded patients who underwent radiotherapy after the first surgery. Preoperative CT and MRI were conducted on all patients, and immediate postoperative CT scans were used in all cases to identify eventual immediate post-surgical complications, while an MRI was routinely used at 3 months for late complications or early recurrence. Follow-ups were conducted with regular post-surgical ambulatory visits, first at 3 months, then at 6 months and finally every 12 months. All surgeries were performed by a senior author (C. Z.). The possible differences between surgeries were minimized considering that all procedures were conducted by the same surgeon with a standardized technique, and lesions characteristics were also comparable (see results). A brief description of the technique is given below.

### Description of the technique

We used a slightly modified superior eyelid crease approach [28]. Patient in general anesthesia, with the head fixed a little over the bed with a Mayfield clamp, allowing gravitational outflow of the irrigation. The sterile surgical field is prepared and neuronavigation is set up. The skin incision on the eyebrow is about 4 cm long, and we aim directly at the superior orbital rim by entering the muscular tissue where the orbitalis blend with the frontalis muscle, reaching the frontal bone. A blunt dissection of the periorbita is performed, and early coagulation of lateral periorbital feeders helps limiting the local blood loss. As the orbital structures are free from their supero-lateral attachments a hand-held retractor is gently set in place to widen up the opening in an infero-medial fashion. As soon as the retractor is posed, the endoscope is inserted. This approach is performed by two surgeons with a four hands technique. At this point, after a check with neuronavigation, the resection of the lesion starts. If the pathology is characterized by a bone involvement and hyperostotic sphenoid wing, the bony part of the resection is performed first. Continuous irrigation is needed to cool down the tip of the drill and to wash away the debris. If the dura is violated it is reconstructed in a standard multilayer fashion with dural substitutes and the help of fibrin glue or other sealants. Once the central part of the surgery is performed, the orbital tissue is posed back in place and a reconstructive suture of the tissues incised is performed. No drain is usually needed.

## Results

In our series, all patients were females, with age ranging between 42 and 78 y.o. Of the five patients, only one had involvement of the orbital apex, three patients had purely superolateral SOMs, and one had superolateral and inferioromedial SOM according to the classification proposed by Agosti et colleagues [1]. All patients had an intraorbital involvement and only one had a spare optic canal. In all cases there was a hyperostosis of the lateral wall of the orbit.

Clinically, all patients presented with proptosis, and three of them had impaired visual acuity. The patient with involvement of the orbital apex showed also palsy of right lateral rectus muscle, and another patient presented with diplopia.

Four of the five patients were previously treated with MTA, while the last patient was previously treated via ETOA. Of all, we dispose data on previous lesion dimension and surgery date of only two patients. Histological examination showed four grade I meningiomas, and one grade II meningioma (WHO 2021). Simpson grade II of resection was obtained in all cases, and none of them underwent adjuvant treatment after the first surgery.

All recurrent lesions were managed with ETOA with the modified superior eyelid crease technique descripted above. Simpson grade I was obtained in all cases, and histological examination remained unchanged in all patients between first and second surgery. After second treatment, the patient with WHO grade II meningioma underwent adjuvant radiation therapy. Time of follow-up varies greatly in our series, between 9 and 84 months. None of the patient had tumor recurrence at last follow-up.

Only the patient with an already compromised function of the right lateral rectus muscle maintained the deficit, while there was no other complication regarding cranial nerve (CN) involvement. Noticeable in our series we had no cerebrospinal fluid (CSF) leaks. All patients showed post-surgical edema of the periorbita, but no other systemic complications were encountered. Furthermore, all patients but one showed proptosis improvement, two of the three patients with visual acuity impairment showed an improvement, and best cosmetic outcome was obtained in all cases. Hospitalization is also reported and varied between 4 and 6 days in our series (see details in Table 1).

**Table 1:** Efficacy and safety of endoscopic modified eyelid crease approach on five patients with recurrent SOMs

Efficacy & safety	Pt 1	Pt 2	Pt 3	Pt 4	Pt 5
Age	78	48	42	50	76
Sex	F	F	F	F	F
Localization*	I, II	I, III	I	I	I
Preop Visual acuity deficit (Yes/No)	yes	yes	no	no	yes
Preop Proptosis (Yes/No)	yes	yes	yes	yes	yes
Others preop symptoms	no	right lateral rectus palsy	diplopia	no	no
Intraorbital involvment (Yes/No)	yes	yes	yes	yes	yes
Cavernous sinus involvement (Yes/No)	no	no	no	no	no
Optic canal involvement (Yes/No)	yes	yes	no	yes	yes
Hyperostosis of lateral wall	yes	yes	yes	yes	yes
Dimension (H x W x L) at first surgery	-	-	2,5 × 2,5 × 2 cm	-	2 × 3 × 3 cm
First surgery date	-	-	02/09/2014	-	04/05/22
First surgery approach**	MTA	MTA	MTA	MTA	ETOA
Hystology after first surgery $	1	1	1	1	2
Simpson grade after first surgery	2	2	2	2	2
Radiotherapy after first surgery (Yes/No)	no	no	no	no	no
Dimension (H x W x L) at second surgery	3,5 × 5,5 × 2 cm	2 × 3 × 2 cm	2 × 2 × 2 cm	3 × 2,5 × 3 cm	1 × 2,3 × 2,8 cm
Second surgery date	31/05/2016	21/09/2016	05/07/2017	05/02/2020	26/08/2023
Second surgery approach**	ETOA	ETOA	ETOA	ETOA	ETOA
Simpson grade after second first surgery	1	1	1	1	1
Hystology after second surgery $	1	1	1	1	2
Radiotherapy after second surgery (Yes/No)	no	no	no	no	yes
Time of followup	60 mo	84 mo	60 mo	36 mo	9 mo
CN focal deficit (Yes/No/temporary)	no	yes	no	no	no
CSF leak (Yes/No)	no	no	no	no	no
Periorbital edema	yes	yes	yes	yes	yes
Other complications	no	no	no	no	no
Vision acuity improvement (Yes/No)	yes	yes	no	no	no
Proptosis improvement (Yes/No)	no	yes	yes	yes	yes
Hospital stay (days)	4	6	5	4	4
Cosmetic outcome £	5	5	5	5	5

* Anatomical class I (lateral or superolateral SOMs), II (medial or inferomedial SOMs), III (orbital apex), IV (diffuse)

**microsurgical transcranial approach (MTA), endoscopic transorbital approach (ETOA)

$ 2021 WHO classification

£patient-reported outcome measures (PROM) (1 = very poor, 2 = poor, 3 = neutral, 4 = good, 5 = excellent)

## Discussion

SOMs have been traditionally treated with MTAs such as pterional or frontotemporal-orbitozygomatic craniotomies [4, 12, 14]. In the last years the role of endoscopic surgery is gaining credibility and there is growing evidence of efficacy and safety of this technique among many skull-base lesions, from the posterior to the anterior cranial fossa [1, 3, 5, 9, 13, 21, 27]. The use of ETOAs for the management of SOMs is growing for the intrinsic characteristics of these lesions that tend to expand towards the sphenoid ridge and often extend in a lateral fashion and determine an hyperostotic reaction of the bone. Furthermore, these lesions tend to enter the orbit from the lateral wall, resulting in the most frequent clinical presentation, that is proptosis [6, 16, 23, 25, 31].

According to a recent systematic review and metanalysis on efficacy and outcomes of the different techniques used for the management of SOMs, best rate results regarding gross total resection (GTR) are obtained with MTAs with 59.8%, while ETOAs rate is 41.3%, and combined ETOAs and EEAs have the lowest rate at 23.5%. The low rate of GTR for combined approaches can be partly explained because EEAs are used for the possibility of reaching medial components of the orbit and the middle cranial fossa. Noticeably GTR is obtained in 78.6% of all anatomical class I lesions (defined as lateral or superolateral SOMs), showing better results compared to lesions that extend to the orbital apex, the cavernous sinus (CS), the superior orbital fissure (SOF), the optic canal (OC) or the anterior clinoid (AC) (GTR rate = 57.3%). Other important factors to be considered are the rate of visual and proptosis improvement, that are higher with the ETOAs compared to MTAs (57.3% and 60% vs. 69.2% and 79.4%). Also, CN focal deficit rate results higher in MTAs (21% vs. 7.3%), although CSF leak rate is similar between MTAs and ETOAs but higher in combined endoscopic techniques (4.9% vs. 5% vs. 20.3% [1].

With the superior eyelid crease approach there is a direct exposure of the supero-lateral aspect of the orbit that allows a short route to the pathological tissue [22, 28], therefore we believe that this technique could be even superior to traditional MTAs in selected cases. It is important to select patients accurately. In our series for example, all patients had a predominant lateral or superolateral growing pattern, with one extending also infero-medially, and another also towards the orbital apex. We believe that best indications for the use of ETOAs in the management of SOMs are: (1) pursuit of total resection in the presence of proptosis or other symptoms due to compression of the intraorbital structures from hyperostotic bone with only a smaller component of the intradural invasion, and in case of (2) meningiomas that are primarily extended along the lateral aspect of the sphenoidal ridge; (3) palliative decompression of the optic nerve in a scenario of symptoms-oriented surgery; (4) as part of a multistaged approach in combination with other techniques, either endoscopic or transcranial [11].

The advantages of this technique are mainly due to the little invasiveness that is necessary to reach deep intraorbital or even middle cranial fossa lesions. MTAs indeed still have intrinsic risks of surgery, such as brain contusion, edema caused by retraction, complications such as epidural or subdural hematomas. Also, large craniotomies often require the use of prosthesis for reconstruction techniques, and calvarial reconstruction following MTAs remains technically challenging [26].

On the other hand, limitations of ETOAs not only are restricted to generic factors that are shared also with MTAs such as the involvement of CS, SOF or OC, but also in case of extremely large lesions that invade the intradural space, it is unwise to proceed with only the endoscopic route. Furthermore, the encasement of intracranial vessels, like the middle cerebral artery, in our opinion represents a strong limitation for the endoscopic treatment.

Overall either surgical or endoscopic techniques result in high complication rates [1] because of the intrinsic complexity of the anatomical area, and the presence of important structures such as CNs or major vessels. Given the morbidity associated with the surgical treatment of these lesions, recently authors have developed a different strategy. Saeed et al. [24] first described the concept of “symptom-oriented” surgery looking for lateral orbit wall decompression rather than aiming for GTR, explaining that SOMs are slow-growing tumors, and subtotal resection (STR) can be the goal of surgery to limit morbidity. In these cases, the role of radiotherapy is essential, and must be conducted in all SOMs that underwent a STR.

In our series, we have analyzed the safety and efficacy of ETOAs for recurrent SOMs. In all cases we reached a Simpson grade I, therefore obtained a GTR through this technique. We had improvement of two of the three patients that had visual acuity impairment, and all but one patient had improvement of proptosis. Complication rate is low in our series. Only one patient had CN deficit that was already present in the preoperatory, and no CSF leaks were observed. All patients showed periorbital edema but was limited to the days after surgery and all obtained a full recovery. We have analyzed cosmetic outcomes using the patient-reported outcome measures (PROM) scale, and all patients reported a score of 5/5. Furthermore, hospitalization ranges between 4 and 6 days, demonstrating that this technique does not only provide good outcomes, but is also safe in terms of complication rates and general recovery. We believe that these outcomes are results of the right selection of the patients. All meningiomas were primarily extended to the lateral aspect of the sphenoid ridge with involvement of the lateral wall of the orbit. Only two cases extended further, one toward the orbital apex, and the other toward the infero-medial aspect of the orbit. Both tumors were resected thanks to gentle dissection and extension to the residual tissue via the dynamic use of the spatula.

This article wants to highlight the importance of having a different strategic option. Indeed, four of the five patients were previously treated with a MTA, and the second surgery performed was an ETOA. Having a virgin route to the pathology is a key factor for the success of the surgery, limiting adhesions and scars of the previous approach. Also, accessing an old surgical scar can lead to secondary complications of the scalp and worse cosmetic results. In a recent article, Zoli et al. [31] conducted a systematic review on the role of ETOAs in SOMs, and described a series of 22 patients, four of which were recurrent SOMs. They found no additional morbidity in any of them. In one case we decided to use an endoscopic approach even if the patient was already treated with an ETOA in the first surgery. We decided to proceed endoscopically because the meningioma was mainly intraorbital with very little extraorbital involvement. In this case we lost the advantage of having a virgin route, but we preferred this approach because given the orbital involvement we thought that an MTA would have been too demolitive and we were confident to achieve a good outcome.

Finally, we believe that ETOAs should always be evaluated as a possible surgical strategy for recurrent SOMs, especially if previously treated with MTAs. The approach must be safe and feasible, therefore careful selection of the patient must be assessed. As we have mentioned before, meningiomas extended to the lateral aspect of the sphenoid ridge with involvement of the lateral wall of the orbit, and with limited involvement of the intradural compartments, are the ideal lesions for this approach. In these cases, we believe that the treatment of choice for recurrent SOMs should be ETOAs rather than MTAs.

We believe that GTR must be pursuit if safe resection can be obtained. We advocate for the use of MTAs for large lesions involving the intradural space and large vessels such as the middle cerebral artery or the carotid artery. Endoscopic approach is not safe in these cases. Also, we think that the surgical choice should reflect the surgeon experience and preference, because the endoscopic techniques have a long learning curve, and surgeons should use what they are more comfortable with. The concept of “symptom-oriented” surgery is interesting and can offer a valid alternative along with adjuvant radiation therapy on the remain pathological tissue.

Limitations of this study include the retrospective nature of the article, the small number and heterogeneity of patients, who were all females, and the lack of a comparison group. For the purpose of our work, we believe that these limitations are of little concern because we primarily wanted to show the feasibility and the main indications for an alternative surgical technique in the management of recurrent SOMs. Clearly, this is a preliminary study, and given the little amount of literature that focuses particularly on recurrent SOMs, further analysis with larger samples can help understand the impact that ETOAs can have on this topic.

## Conclusion

Endoscopic surgery is showing large application for skull-base tumors, and this is particularly true regarding SOMs. ETOAs show great outcomes in terms of visual acuity and proptosis improvement. Also, CN deficits are low and CSF leak rates are comparable to those treated with MTAs. This technique is also characterized by a quick recovery and a short hospitalization time. We support the use of ETOAs for the management of recurrent SOMs in selected cases involving primarily the intraorbital compartment and the lateral orbital wall, with little intradural expansion. When previously treated with MTAs, a recurrent lesion, if feasible, should be managed via endoscopic technique, allowing the surgeon to use a virgin route to the pathological tissue.

## Data Availability

No datasets were generated or analysed during the current study.
